# IurV, Encoded by ORF VCA0231, Is Involved in the Regulation of Iron Uptake Genes in *Vibrio cholerae*

**DOI:** 10.3390/genes11101184

**Published:** 2020-10-12

**Authors:** Bernardo Sachman-Ruiz, José Antonio Ibarra, Paulina Estrada-de los Santos, Alexia Torres Muñoz, Begoña Giménez, Juan Carlos Salazar, Víctor Antonio García-Angulo

**Affiliations:** 1 Programa de Microbiología y Micología, Instituto de Ciencias Biomédicas, Facultad de Medicina, University of Chile, 8380453 Santiago, Chile; sachman.bernardo@inifap.gob.mx (B.S.-R.); ale.ntm6@gmail.com (A.T.M.); jcsalazar@u.uchile.cl (J.C.S.); 2 Centro Nacional de Investigacion Disciplinaria en Salud Animal e Inocuidad del Instituto Nacional de Investigaciones Forestales, Agrícolas y Pecuarias, 62574 Morelos, Mexico; 3 Departamento de Microbiología, Escuela Nacional de Ciencias Biológicas, Instituto Politécnico Nacional, 11340 Mexico, Mexico; jaig19@gmail.com (J.A.I.); pestradadelossantos@gmail.com (P.E.-d.l.S.); 4 Departamento de Ciencia y Tecnología de los Alimentos, Facultad Tecnológica, Universidad de Santiago de Chile, 9170124 Santiago, Chile; bego.gimenez@usach.cl

**Keywords:** iron, *Vibrio cholerae*, transcriptomics, AraC/XylS, genetic context

## Abstract

The pathogen *Vibrio cholerae* has multiple iron acquisition systems which allow bacteria to exploit a variety of iron sources across the different environments on which it thrives. The expression of such iron uptake systems is highly regulated, mainly by the master iron homeostasis regulator Fur but also by other mechanisms. Recently, we documented that the expression of many of the iron-responsive genes is also modulated by riboflavin. Among them, the open reading frame VCA0231, repressed both by riboflavin and iron, encodes a putative transcriptional regulator of the AraC/XylS family. Nonetheless, the genes or functions affected by this factor are unknown. In the present study, a series of in silico analyses was performed in order to identify the putative functions associated with the product of VCA0231. The STRING database predicted many iron uptake genes as functional partners for the product of VCA0231. In addition, a genomic neighborhood analysis with the Enzyme Function Initiative tools detected many Pfam families involved in iron homeostasis genetically associated with VCA0231. Moreover, a phylogenetic tree showed that other AraC/XylS members known to regulate siderophore utilization in bacteria clustered together and the product of VCA0231 localized in this cluster. This suggested that the product of VCA0231, here named IurV, is involved in the regulation of iron uptake processes. RNAseq was performed to determine the transcriptional effects of a deletion in VCA0231. A total of 52 genes were overexpressed and 21 genes were downregulated in response to the *iurV* deletion. Among these, several iron uptake genes and other iron homeostasis-related genes were found. Six gene ontology (GO) functional terms were enriched in the upregulated genes, of which five were related to iron metabolism. The regulatory pattern observed in the transcriptomics of a subset of genes was independently confirmed by quantitative real time PCR analysis. The results indicate that IurV is a novel regulator of the AraC/XylS family involved in the repression of iron uptake genes. Whether this effect is direct or indirect remains to be determined.

## 1. Introduction

*V. cholerae* is the causative agent of cholera, a pandemic disease characterized by acute watery diarrhea. This bacterium causes around 4.3 million cholera cases leading to 143,000 deaths globally each year [1]. Once the bacterium is ingested, cholera develops after the expression of the cholera toxin (CT) in the human digestive tract by toxigenic strains of *V. cholerae* and may cause rapid dehydration producing hypovolemic shock, acidosis and death within hours if untreated [2]. *V. cholerae* is a mostly innocuous aquatic species. Cholera epidemics are caused by a narrow group of strains belonging mainly to serogroups O1 and O139, which conserve the CT genes [3]. Notably, temporal instability in social fabric may lead to the development of outbreaks, such as the cases of the Haiti outbreak that followed the 2010 earthquake and the ongoing Yemen outbreak that started in 2016 after the eruption of the civil war [4,5]. In addition, it has been postulated that global warming is an important factor for the increase in cholera cases worldwide [6]. All these factors make the study of the physiology of *V. cholerae* critical in global public health.

This bacterium can subsist in diverse environments from sea and estuarine environments to the human intestinal tract [3]. For this, *V. cholerae* must withstand constant changes in nutrient availability and needs to adapt to those different niches. A critical micronutrient for this species, as for most bacteria, is iron. This metal is required for a myriad of cellular processes, mainly due to its redox properties [7]. Although this is one of the most common elements on earth, environmental iron is usually present in its oxidized, insoluble form [7,8]. Moreover, within hosts, iron is trapped by binding proteins [9]. Hence, *V. cholerae* must deal with low iron concentrations both in the environment and during host colonization. The genome of this species is abounding in genes for iron uptake systems that allow the acquisition of a variety of iron forms [10]. The genes encoded by the *vib* operon synthesize the siderophore vibriobactin that scavenges extracellular Fe^3+^, which is further internalized after its interaction with its receptor ViuA, the product of the *viu* gene [11,12,13]. Either the ViuPDGC or the VctPDGC system can transport the iron-bound siderophore towards the cytoplasm [13,14,15]. This pathogen is also able to uptake xenosiderophores (siderophores produced by other species) using receptors such as IrgA [16]. In addition to its role in siderophore uptake, the VctPDGC system is involved in the direct uptake of free iron [10,14,15]. Within hosts, the heme group can be an important source of iron, which *V. cholerae* acquires through the outer membrane receptor HutA and the HutBCD inner membrane transport complex. These systems, as well as the siderophore uptake systems, are dependent on the inner membrane TonB/ExbDE energy transduction complex. Two TonB/ExbDE systems are encoded in *V. cholerae* [14,17,18,19,20]. In addition, free extracellular ferric iron can be internalized by the ATP binding cassette-type FbpABC importer and ferrous iron by the FeoABC transport system [10,21]. The expression of the iron uptake systems is tightly regulated, as excessive iron is detrimental due to the formation of reactive oxygen species. The master iron homeostasis regulator is the ferric uptake regulator (Fur). The interaction of this regulator with iron under iron-replete conditions prompts its activity as repressor by binding to specific DNA sequences named Fur boxes [22,23]. Iron uptake is also under the regulation of other factors such as RyhB, a Fur-repressed small RNA required for posttranscriptional regulation of iron internalization, and other genes involved in iron homeostasis [14,24,25]. In addition, in other bacterial species, the role of transcriptional factors of the AraC/XylS family in the regulation of the expression of genes involved in siderophore biosynthesis and uptake has been documented. YbtA activates the expression of the yersiniabactin biosynthesis operon and the siderophore receptor in *Yersinia pestis* and some strains of pathogenic *Escherichia coli* [26,27,28]. PchR regulates the transcription of the siderophore pyochelin receptor in *Pseudomonas aeruginosa* [29,30]. AlcR activates alcaligin biosynthesis and uptake in *Bordetella* species [31,32]. In *Neisseria gonorrhoeae*, MpeR activates *fetA*, encoding the receptor of the enterobactin xenosiderophore [33]. Finally, DesR activates the transcription of *desA,* coding for the xenosiderophore ferrioxamine B receptor in *Vibrio vulnificus* and *Vibrio furnissi* [34,35] and *Aeromonas hydrophila* [36].

The iron regulatory circuit ensures that iron acquisition and other iron-related functions are coordinately expressed in response to iron levels. In some bacteria, iron shortage also affects the expression of riboflavin biosynthesis and uptake genes. It has been hypothesized that increasing riboflavin pools helps in surpassing iron scarcity conditions because riboflavin may reduce extracellular Fe^3+^ into Fe^2+^, which augments iron solubility. In addition, rising riboflavin production may boost flavoproteins required for iron uptake and prompt the replacement of ferredoxins by flavodoxins [37]. In *V. cholerae*, we recently documented that the riboflavin regulon determined by transcriptomics highly overlaps with that of iron. Riboflavin represses most of the iron uptake systems and other iron-regulated genes. Notably, among the genes regulated both by iron and riboflavin is the open reading frame (ORF) VCA0231, encoding a transcriptional regulator of the AraC/XylS family [38]. In the *V. cholerae* genome, VCA0231 is encoded between the gene for the siderophore enterobactin receptor *vctA* and the siderophore utilization operon *vctPDGC* [16]. VCA0231 was recently shown to be induced in the digestive tract of an animal model of *V. cholerae* infection, together with genes involved in iron uptake and other iron-repressed genes [39]. Nonetheless, the function of VCA0231 is still unknown. In the current study, genomic context of the VCA0231 ORF and phylogenetic analysis of its product were performed in order to gain insights into its putative regulatory functions. In addition, a transcriptomics approach was used to identify the genes responding to VCA0231 in *V. cholerae*.

## 2. Materials and Methods

### 2.1. STRING and EFI-GNT Analysis

The ORF VCA0231 from *V. cholerae* N16961 (VC_A0231) was queried in the STRING database (https://string-db.org/) [40]. The protein–protein interaction networks map and the predicted functional partners table were retrieved. For the EFI-GNT analysis [41], the amino acid sequence of VCA0231 from *V. cholerae* N16961 was retrieved from the Kyoto Encyclopedia of Genes and Genomes (https://www.genome.jp/kegg/) and used to generate a sequence similarity network (SSN) in the EFI Enzyme similarity tool (University of Illinois, Urbana-Champaign, IL, USA) (https://efi.igb.illinois.edu/efi-est/). For this SSN, the UniProt BLAST query e-value was set to 13. The SSN was submitted to the EFI Genome neighborhood tool (University of Illinois, Urbana-Champaign, IL, USA) (https://efi.igb.illinois.edu/efi-gnt/). For this analysis, a genome neighborhood size of 3 and a minimal Pfam co-ocurrence percentage of 40% (twice the stringency of the default value) were set. The SSN, Pfam associations and the genetic neighborhoods results were visualized using the Cytoscape software (The Citoscape Consortium, San Diego, CA, USA).

### 2.2. Phylogenetic Trees Construction

A protein alignment of the 58 AraC/XylS transcription factors used in the report by Ibarra et al. [42] together with the amino acid sequences for YbtA (Q56951), AlcR (ETG99811.1), MpeR (WP_003692470.1), DesR (WP_011705822.1), PchR (P40883) and the product of VCA0231 was performed with Muscle 3.57 (drive5, Mill Valley, CA, USA) [43]. A second alignment was done using only the DNA binding domain of these proteins. The phylogenetic trees were constructed with the maximum likelihood (ML) method using PAM250 matrix with the program PhyML (Montpellier Bioinformatics Platform, Montpellier, France) [44]. The trees were visualized using Mega v7 (Tokyo Metropolitan University, Tokyo, Japan) [45].

### 2.3. Construction of a V. cholerae ΔVCA0231::cat Strain

A *V. cholerae* ΔVCA0231::*cat* was constructed by homologous recombination with a PCR fragment containing a chloramphenicol acetyl transferase gene (*cat*) as previously described [46]. Briefly, *V. cholerae* was electroporated with pSIM19 and transformants selected in lisogeny broth (LB) plates plus spectinomycin (100 µg/mL) at 30 °C. One transformant was grown in super optimal broth (SOB) medium at 30 °C to an optical density (O.D._600nm_) equal to 0.4. Next, the culture was incubated at 42 °C for 20 min to induce the expression of the recombination system from pSIM19. Cells were centrifuged and washed three times with distilled water and electroporated with a PCR product obtained with primers araC H1P1 and araC H2P2 (Supplemental Table S1) and plasmid pCLF3 [47] as template. Such a PCR fragment has a *cat* gene flanked by 39 bp homologous to the 5′ and 3′ end regions upstream and downstream of VCA0231. Candidate mutants were selected on LB plus chloramphenicol (5 µg/mL) at 42 °C. Candidates were screened for the replacement of the VCA0231 ORF by the *cat* gene by PCR with primers flanking the mutation site araC Fw and araC Rv (Supplemental Table S1). Correct junction sites were further corroborated with the set of primers araC Fw/catRv (amplifying 5′ junction) and ara C Rv/catFw (3′ junction). Overall, this mutation is an internal deletion that substitutes the coding region of VCA0231 from codons 4 to 354 for a chloramphenicol resistance cassette. The upstream gene *vctC* is convergent to VCA0231 and the downstream gene is encoded divergently from VCA0231. Thus, this internal deletion does not produce polar effects.

### 2.4. Strains Culture and RNA Extractions

*V. cholerae* N16961 and its isogenic ΔVCA0231::*cat* derivative strain were grown overnight in LB plates at 37 °C. Two tubes with 5 mL of LB each were inoculated with an isolated colony from the plate cultures and incubated at 37 °C in an orbital shaker at 150 rpm overnight, with 5 µg/mL chloramphenicol added to the mutant culture. Next, 1 mL of each culture was centrifuged, the pellets were washed twice with M9 minimal medium and resuspended in 30 mL of fresh M9 and incubated at 37 °C and 150 rpm until an O.D._600nm_ of 0.5. One milliliter of each culture was centrifuged and subjected to RNA extraction with the GeneJet RNA purification kit (Thermo Scientific, Waltham, MA, USA) according to the manufacturer’s instructions. The same growth conditions and RNA isolation protocols were applied for RNA subjected to both transcriptomics and quantitative real time-polymerase chain reaction (qRT-PCR).

### 2.5. qRT-PCR

All RNA samples were treated with DNAse (Invitrogen, Carlsbad, CA, USA) according to the manufacturer´s instructions. AffinityScript QPCR cDNA Synthesis kit (Agilent Technologies, Santa Clara, CA, USA) was used for cDNA synthesis according to manufacturer’s instructions. qRT-PCR was performed using the Brilliant II SYBR Green QPCR Master Mix kit in a Mic qPCR Cycler (Bio Molecular Systems, Upper Coomera, Australia). For each qRT-PCR run, a negative control reaction for each primer pair using RNA with no reverse transcriptase treatment was included. Relative expression between the indicated conditions was determined through the ΔΔCt method as developed before [48]. The 16S ribosomal RNA gene was used for normalization, using the set of primers 16S Fw/16S Rv [49]. For *ribA*, *ribN, ribD* and *tonB*, the sets of primers used were ribA Fw/ribA Rv, ribN Fw/ribN Rv, ribD Fw/ribD Rv and tonB Fw/tonB Rv, respectively [49]. For the *fur*, *sodA*, *vctA* and *vctC* genes, the set of primers used for qRT-PCR analysis were fur Fw/fur Rv, SODFe Fw/SODFe Rv, vctA Fw/vctA Rv and vctC Fw/vctC R, respectively. The sequence of these primers is shown in Supplemental Table S1.

### 2.6. RNAseq Analysis

Ribosomal RNA was depleted with the NEBNext Bacterial rRNA depletion kit (New England Biolabs, Ipswich, MA, USA) and RNA was reverse transcribed and sequenced at Macrogen (Korea), with the Illumina HiSeq platform(Illumina Inc, San Diego, CA, USA) to produce 100 bp paired-end reads, with ~40 million reads per sample. Three independent libraries were constructed and sequenced for each strain. Differentially expressed genes were determined in the PATRIC platform (University of Chicago, Chicago, IL, USA) using the Tuxedo strategy and *V. cholerae* N16961 as target genome [50]. Reads were aligned using Bowtie2 [51], assembled with Cufflinks and differential expression was detected by CuffDiff [52]. Genes with at least a three-fold change in the mutant were considered differentially expressed. All the selected genes had statistical difference (*p* < 0.01). Gene ontology (GO) terms enrichment analyses using the PANTHER classification system was performed on the platform of the Gene Ontology Consortium (www.geneontology.org) [53,54], and statistically overrepresented (*p* < 0.01) functional terms at the biological process level were retrieved. Original RNAseq data were deposited on the PATRIC database under the public workspace named *VCA0231*.

## 3. Results

**Figure 1:**
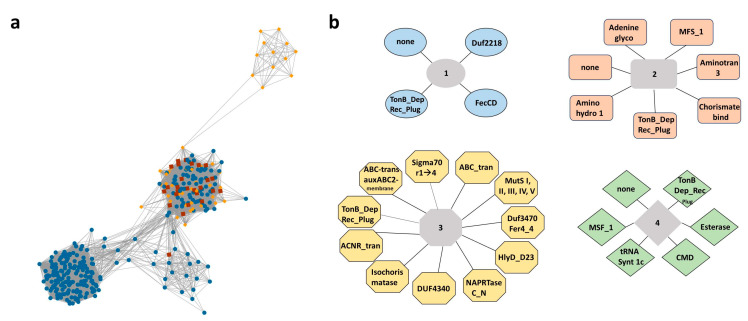
Genome neighborhood analysis of VCA0231. (**a**) Sequence similarity network (SSN) constructed for the product of VCA0231 using the enzyme similarity tool from EFI. Nodes represent protein orthologs of the product of VCA0231 in species from α-Proteobacteria (blue octagons), β-Proteobacteria (red rectangles) and γ-Proteobacteria (yellow circles). (**b**) Pfam families associated with each cluster of the SSN, as determined by GNT.

The ORF VCA0231 is part of the set of iron-regulated genes that are repressed by riboflavin in *V. cholerae*s [22,38]. This gene codes for a putative protein of 358 amino acid residues conserving a helix-turn-helix (HTH) domain of the AraC/XylS type in its amino terminal region (Supplemental Figure S1). In order to identify putative functions for VCA0231, we initially searched for this gene in the STRING database, which predicts functional partners by information collected from known experimental and in silico functional interaction model data [40]. STRING identified ten predicted functional partners for the product of VCA0231 (Supplemental Figure S2 and Supplemental Table S2). Out of these, six are proteins involved in iron acquisition: FhuA and ViuA, which are siderophore receptors, VC1579 and VibB, two siderophore biosynthetic proteins, TonB, required for the ATP hydrolysis-driven energization of various iron uptake systems, and IrgB, an activator of the expression of the IrgA enterobactin receptor. These functional partners were identified in the database mostly on the basis of genetic neighborhood, co-occurrence, co-expression and text mining (Supplemental Table S1). The genomic localization of VCA0231 in *V. cholerae* suggests its involvement in regulation of siderophore utilization genes. To get insights into the function of VCA0231 from other bacteria, we performed an analysis of genomic context of VCA0231 orthologs across bacterial genomes using the Genome Neighborhood Tool of the Enzyme Function Initiative (EFI-GNT). In such an approach, a sequence similarity network cluster is determined for a quest protein and the genetic neighborhood of each member is screened to determine Pfam families statistically associated with each cluster [41,55,56]. For VCA0231, a total of 348 orthologs organized in four clusters, distributed in α-, β- and γ-proteobacteria, were found (Figure 1a). Four Pfam families were associated with cluster 1, seven with cluster 2, eleven with cluster 3 and six with cluster 4 (Figure 1b). The TonB-dependent receptor plug domain was commonly linked to all clusters. This is a domain that acts as a channel gate conserved in TonB-dependent outer membrane receptors [57]. In all four clusters, genes containing this Pfam family corresponded to siderophore receptors, which are known to depend on TonB for their uptake activity. Other Pfam families associated with the clusters were also present in genes known to be required for iron uptake in bacteria. Most of the genes containing the FecCD and the ABC transport Pfams corresponded respectively to permease and ATPase components of ABC transport systems predicted to uptake different siderophores such as vibriobactin, enterobactin, anguibactin and ferrichrome, or other iron sources such as ferric iron of ferric dicytrate. The genes containing the Pfam chorismate binding enzyme corresponded to isochorismate synthase genes involved in siderophore bisyntesis. The isochorismatase Pfam is found in proteins involved in siderophore biosynthesis. Finally, the Fer4_4 Pfam is present in ferredoxins. Thus, the results from STRING and EFI-GNT analysis strongly suggest that VCA0231 participates in the regulation of iron homeostasis-related genes in *V. cholerae*.

**Figure 2:**
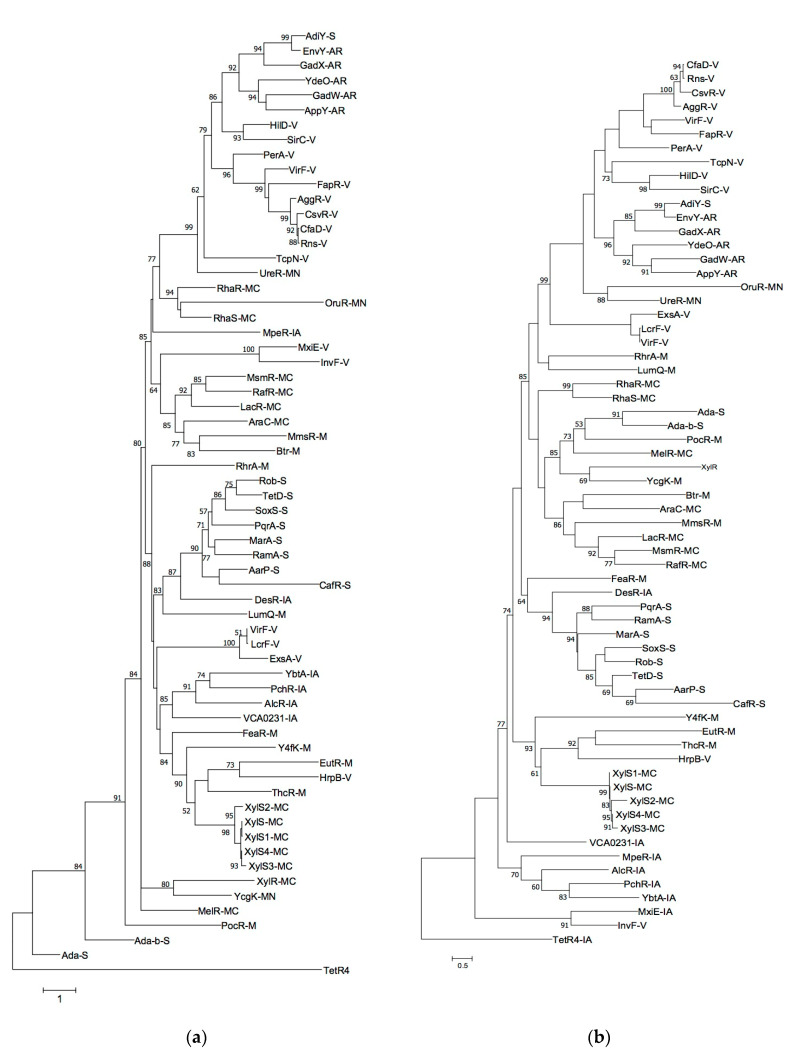
Phylogenetic analysis of representative AraC/XylS transcriptional regulators of different physiological functions. A phylogenetic tree of well-characterized AraC/XylS transcriptional factors was constructed with an alignment of the whole protein (**a**) or the DNA binding domain (**b**) of the 58 AraC/XylS transcription factors used in the report by [42] together with YbtA, PchR, AlcR, MpeR, DesR and the product of VCA0231. Numbers in each branch represent the bootstrap values of 1000 replicates. Broad functional categories are indicated by capital letters as a suffix as follows: general metabolism (M); metabolism of carbon sources (MC); utilization of nitrogen sources (MN); adaptive responses (AR); stress responses (S); virulence (V) and iron acquisition (IA). The bottom ruler bar in each panel indicates number of substitutions per position.

The members of the AraC/XylS family conserve two HTH motifs usually at the C terminal domain that interacts with the DNA to exert their transcriptional regulatory activity [58,59]. In general, the N terminal is composed of an effector domain which is more variable among the members of the family. This domain mediates specific cofactor binding and occasionally also multimerization. Notably, AraC/XylS regulators may form clade clustering members involved in the regulation of the same specific physiological functions, namely general metabolism, catabolism of carbon sources, utilization of nitrogen sources, adaptive and stress response and virulence [42]. This property has allowed functional prediction for uncharacterized members of the family. Although AraC/XylS regulators are increasingly being reported to regulate iron acquisition in different bacterial species, is not known if such regulators form a distinctive phylogenetic cluster among the AraC/XylS members with different functions. Thus, a phylogenetic tree with the sequences of selected AraC/XylS regulators with different functions used in a previous study [42] and the known bacterial AraC/XylS regulators of iron acquisition YbtA, PchR, MpeR, AlcR and DesR plus the product of VCA0231 were constructed. As reported before, Figure 2a shows that with a few exceptions, AraC/XylS regulators form clusters of regulators with a similar physiological function. Likewise, most of the iron uptake regulators formed a cluster, including YbtA, PchR and AlcR in a branch and VCA0231 in another branch. Within this phylogeny, MpeR constitutes an independent branch, while DesR localized in a mixed cluster that included regulators of differing functions such as sulfur utilization, virulence and general metabolism. The results suggest that AraC/XylS regulators involved in iron acquisition tend to form a cluster, as described for those with other functions. The product of VCA0231 and its orthologs may represent a subfamily of AraC/XylS regulators of iron acquisition. When a phylogenetic tree using only the highly conserved C terminal segment containing the DNA binding domain was constructed, again most of the iron-acquisition regulators were grouped within a clade (Figure 2b). Nonetheless, VCA0231 formed an independent, single-member clade. This suggests that for this regulator, the DNA binding domain evolved differently to those of the other AraC/XylS transcription factors involved in iron acquisition. Overall, phylogenetic analysis supported the hypothesis that the product of VCA0231 regulates iron acquisition in *V*. *cholerae* and was named IurV (iron uptake regulator in *V. cholerae*).

In order to experimentally determine the physiological functions in which IurV is involved, a VCA0231 null mutant was constructed in *V. cholerae*. Then, RNAseq was performed to identify genes whose expression is affected by the mutation. In total, 73 transcriptional units had differential expression according to the parameters used (Table 1). Of these, 52 genes were overexpressed and 21 were repressed in response to *iurV* deletion. In the group of the overexpressed genes, most are involved in iron uptake and storage and in the metabolism of carboxylic acids. Among the iron homeostasis genes, systems for ferrous and ferric iron uptake and heme and siderophore biosynthesis and utilization systems are included. An analysis of enrichment of Gene Ontology (GO) terms in this subset of genes using the PANTHER classification system [53,54] identified six biological processes overrepresented: vibriobactin biosynthetic process, siderophore biosynthetic process, siderophore transport, cellular iron ion homeostasis, iron ion transmembrane transport and monocarboxylic acid transport. In the set of genes with reduced expression, a periplasmic and two membrane protein genes were amongst the most repressed. Two hemolysin genes and genes involved in amino acid metabolism, transport, oxidative stress and redox metabolism were also found. Notably, a putative ferric reductase was also repressed. This suggests that some genes related to iron and redox metabolism are also downregulated in the *iurV* mutant. Nonetheless, the GO enrichment analysis did not find any particular term overrepresented in this group.

**Figure 3:**
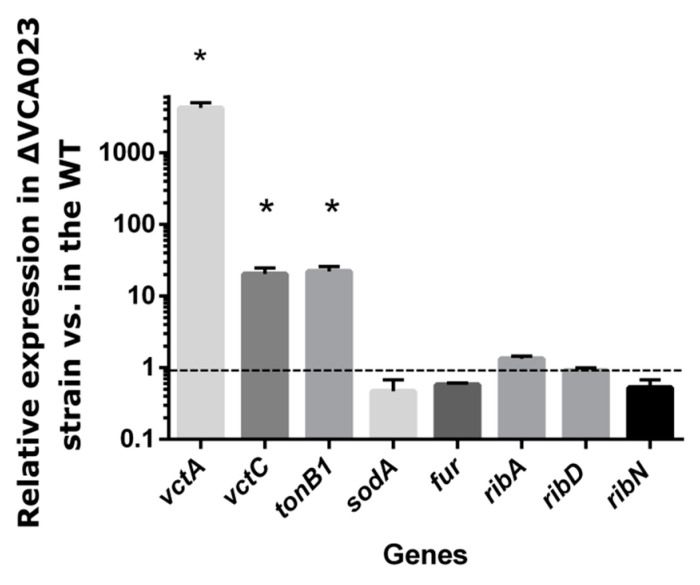
Relative gene expression in the VCA0231 mutant determined by qRT-PCR. *V. cholerae* WT and its ΔVCA0231::*cat* derivative were grown in M9 media and RNA extracted and qRT-PCR performed to determine the relative expression of the indicated genes. The results shown are the average and standard deviation of three independent experiments. Statistically significant differences (*p* < 0.05) are indicated by asterisks.

The siderophore enterobactin receptor *vctA* gene, encoded divergently to *iurV*, was the most upregulated gene in the mutant, with 409-fold change in expression with respect to the WT. Genes of the *vctPDGC* operon encoded convergently to *iurV*, which are required for the uptake of the enterobactin and vibriobactin siderophores, were also found overexpressed by transcriptomics. In order to independently confirm these results, we used qRT-PCR to determine the relative expression of the *vctA* and *vctC* genes in the ΔVCA0231::*cat* vs. the WT strain, using the 16S rRNA subunit gene as internal normalizer [48]. For this, we applied the results in Figure 3 to show that the expression of these two genes is significantly increased in the mutant. Moreover, qRT-PCR also confirmed that *tonB1* is overexpressed. Thus, both the transcriptomics and qRT-PCR results indicate that *iurV* is involved in the regulation of iron uptake genes. Most of the genes with differential expression in the *iurV* mutant are known to be upregulated in low iron [22]. Nonetheless, other genes also known to be induced in low iron conditions were not responsive to *iurV* according to the transcriptomics results. For example, the gene *sodA*, coding for a superoxide dismutase, is one of the most induced genes in the absence of iron [22], but it was not present in the set of genes regulated by *iurV* determined by our transcriptomics data. qRT-PCR confirmed that the expression of this gene is unaffected by the *iurV* deletion (Figure 3). Thus, it seems that *iurV* participates in the regulation of a subset of the iron-regulated genes, mostly those involved in iron uptake. Fur, the global regulator of iron homeostasis, is not regulated by IurV according to transcriptomics and no significant changes in its expression were detected by qRT-PCR (Figure 3), further discarding the possibility that *iurV* acts through the regulation of *fur*.

We have previously shown that riboflavin and iron reciprocally regulate their provision genes [38]. Given that *iurV* is regulated by riboflavin, this gene is a good candidate to be involved in the regulation of riboflavin provision genes. Nonetheless, neither riboflavin biosynthetic genes nor the *ribN* gene encoding the riboflavin importer [60] were regulated in the transcriptomics experiment. The expression of the biosynthetic genes *ribA*, *ribD* and of the transporter-encoding gene *ribN* was further assessed by qRT-PCR (Figure 3). No statistical difference was detected in the ratio of expression between the *iurV* and WT strains, confirming that *iurV* is not involved in the regulation of riboflavin supply pathways under these conditions.

## 4. Discussion

Iron metabolism is a highly regulated trait in bacteria, as excessive iron is pernicious for viability. Fur, which binds iron to repress the transcription of iron transport genes and the RyhB small regulatory RNA which downregulates the expression of iron-dependent enzymes, are among the most important iron homeostasis regulatory factors in *V. cholerae* [14,24,61]. While the regulatory circuit involving Fur and RyhB is conserved in several bacteria [23,62], some iron regulatory cues are more restricted to certain genera. These include iron-dependent regulation of genetic expression using AraC/XylS transcription factors [26,30]. The genomic localization of VCA0231, together with its co-expression with iron-related features determined in previous works [16,38,39], suggested its participation in the regulation of iron internalization in *V. cholerae*. Genomic context is a widely used tool to infer genetic functions [63]. In this study, orthologs of *iurV* were shown to colocalize with genes coding for proteins belonging to Pfam families related to iron internalization. IurV orthologs were found in α-, β- and γ-proteobacterial species, suggesting that IurV represents a novel family of AraC/XylsS regulators of iron uptake in a subset of proteobacteria. Transcriptomics and qRT-PCR corroborated the role of IurV in the regulation of iron uptake systems. Five out of the six biological processes enriched in the GO terms of genes upregulated are related to iron metabolism. Our transcriptional study revealed that IurV is involved in the repression of most of the iron uptake systems in this species. The AraC/XylS regulators YbtA, PchR and MpeR directly activate the transcription of iron uptake genes in other bacteria, particularly siderophore and siderophore receptors [26,33,64]. Thus, an important difference of IurV with other cases is that this regulator acts to repress iron uptake. Nonetheless, if this effect is direct or indirect (i.e., through direct repression of an activator of iron uptake genes, or alternatively, activation of a repressor) remains the subject of future work. Published reports indicated that at least some iron-related AraC/XylS regulators could act both as activators and repressors after DNA binding, depending on the operator. For example, in addition to its role as activator of the *fetA* gene, MpeR is able to directly repress *mtrR*, which encodes a repressor of the efflux pump operon *mtrCDE* in *N. gonorrhoeae* [65]. Additionally, PchR is known to differentially act as an activator over the *ftpA* gene and a repressor of its own gene [64]. In our transcriptomics study, the ORF VCA0029, coding for a transcriptional regulator of the IclR family, was repressed 5.6 times in the *iurV* mutant. The probable role of this regulator as mediator of the effect of IurV is worth evaluating. Noteworthy, VCA0231 itself is known to be repressed by Fur [22]. Thus, this regulatory cascade acts downstream of the master iron regulator, similarly to what has been described for MpeR [66].

Although no GO terms were found to be enriched in the set of genes repressed in response to the *iurV* mutation, some features related to iron homeostasis could be recognized. Notably, two genes encoding hemolysins are included in this group. The role of hemolysis as a means to increase iron bioavailability has been hypothesized before [37]. In addition, genes for a thioredoxin 2 and a glutaredoxin, both enzyme types involved in electron transfer, are also repressed. Iron is required mostly because of its redox properties; thus, changes in iron uptake are expected to change the general redox metabolic status. Also among the genes repressed was VC2215, coding for an ATPase required in a heavy metal transport system. Similarly, MpeR is involved in the regulation of tellurium resistance genes [65]. Thus, it could be speculated that common traits co-regulated with iron uptake may be integrated by the use of AraC/XylS transcriptional regulators in bacteria. In addition to iron-related biological processes, the term *monocarboxylic acid transport* was enriched in the group of genes upregulated in the *iurV* mutant. Hence, this regulator may also be involved in the modulation of other metabolic traits, both dependently and independently of its effect on iron uptake.

Together with iron, riboflavin-derived molecules are the most important redox cofactors in enzymatic reactions [67]. Probably owing to this common property, iron and riboflavin metabolism has been reported to be highly related in some bacteria [37,38,68,69,70,71,72]. In *V. cholerae*, we have documented that riboflavin and iron reciprocally regulate their provision systems [38]. These regulatory effects are coordinated by the presence of iron and riboflavin in the media. We speculated that IurV may be a candidate to integrate regulatory responses to iron and riboflavin in this species. However, riboflavin provision genes were unaffected by the elimination of *iurV*, and this result was further confirmed by qRT-PCR on the *ribD*, *ribA* and *ribN* genes. This suggests that *iruV* is not involved in the cross-regulation between iron and riboflavin. Nonetheless, evaluation of its effects on different conditions of iron and riboflavin availability is necessary in order to confirm this.

## 5. Conclusions

By combining in silico and experimental approaches, this study identified iron internalization pathways as the regulatory target of IurV encoded by the VCA0231 ORF in *V. cholerae*. This protein may represent a novel family of iron uptake regulators conserved in α-, β- and γ-proteobacteria. Its mechanism of regulation and further role in other bacteria remains to be investigated.

## Figures and Tables

**Table 1:** Differentially expressed genes in response to the VCA0231 elimination in *V. cholerae*.

ORF	Gene Name	Log2 Fold Change	*p* Value	Annotation
VC2691	*cpxP*	−5.95515	<5.00E−05	P pilus assembly/Cpx signaling pathway, periplasmic inhibitor/zinc−resistance associated protein
VC0633	*OmpU*	−5.92287	<5.00E−05	Outer membrane porin OmpU
VCA0261−0		−5.08589	<5.00E−05	Hypotetical protein, Hypotetical protein
VCA0262		−3.93658	0.0024	Hypotetical protein
VC1587		−3.42537	<5.00E−05	Membrane protein
VCA0615	*msrB*	−3.27937	<5.00E−05	Peptide−methionine (S)−S−oxide reductase MsrA/Peptide−methionine (R)−S−oxide reductase MsrB
VCA0258		−3.22844	<5.00E−05	Hemolysin
VC2216	*copG*	−2.95846	<5.00E−05	CopG protein
VCA0650−1		−2.94231	<5.00E−05	D−alanine−D−alanine ligase, Hipotetical protein
VCA0139		−2.84816	<5.00E−05	Acyl−CoA synthetase
VC2215		−2.5293	<5.00E−05	Lead, cadmium, zinc and mercury transporting ATPase; Copper−translocating P−type ATPase
VCA0029		−2.48946	<5.00E−05	Transcriptional regulator, IclR family
VC0232		−2.40928	<5.00E−05	Hypotetical protein
VCA0752		−2.16805	<5.00E−05	Thioredoxin 2
VCA0648−9		−2.08498	<5.00E−05	Hypotetical protein, Hypotetical protein
VCA1011		−2.07667	<5.00E−05	Glutaredoxin
VC1044−5		−2.00207	<5.00E−05	Hypotetical protein, RNA polymerase ECF−type sigma factor
VCA0646−7		−1.97651	<5.00E−05	Putative hemolysin
VC1723		−1.88711	<5.00E−05	COG0398: uncharacterized membrane protein
VC0143		−1.88157	<5.00E−05	Hypotetical protein
VCA0151		−1.72402	<5.00E−05	Predicted ferric reductase
VC0285		1.68838	<5.00E−05	4−hydroxy−2−oxoglutarate aldolase; 2−dehydro−3−deoxyphosphogluconate aldolase
VCA0687	*fbpC*	1.75742	<5.00E−05	ABC transporter, ATP−binding protein (cluster 1, maltose/g3p/polyamine/iron); ABC transporter, ATP−binding protein (cluster 10, nitrate/sulfonate/bicarbonate) | fbpC
VC1589		1.76693	<5.00E−05	α−acetolactate decarboxylase
VC2076−77−78	*feoABC*	1.81165	<5.00E−05	Ferrous iron transport protein C, Ferrous iron transporter FeoB, Ferrous iron transporter−associated protein FeoA
VCA0686		1.82212	<5.00E−05	ABC transporter, permease protein 1 (cluster 1, maltose/g3p/polyamine/iron)/ABC transporter, permease protein 2 (cluster 1, maltose/g3p/polyamine/iron)
VC1334		1.82445	<5.00E−05	Tripartite tricarboxylate transporter TctC family
VC1266−7		1.84393	<5.00E−05	Iron−regulated protein A precursor, Hypotetical protein
VC2761		1.84679	<5.00E−05	Multidrug resistance transporter, Bcr/CflA family
VC1543−44−45−46	*exbB2* *exbD2* *tonB2* *VC1543*	1.88124	<5.00E−05	TPR domain protein, putative component of TonB system, putative TonB−dependent receptor, Ferric siderophore transport system, biopolymer transport protein ExbB
VC0284		2.02153	<5.00E−05	Ferrichrome−iron receptor
VCA1041		2.0355	<5.00E−05	Phosphosugar mutase of unknown sugar (see annotation)
VC1572		2.12029	<5.00E−05	Hypotetical protein
VC1265		2.15881	<5.00E−05	Probable thiol oxidoreductase with 2 cytochrome c heme−binding sites
VC0364	*bfd*	2.22671	<5.00E−05	Bacterioferritin−associated ferredoxin
VC0286	*gntU*	2.22969	<5.00E−05	Low−affinity gluconate/H+ symporter GntU
VCA0976		2.23539	<5.00E−05	Hypotetical protein
VCA0984	*ildD*	2.27194	<5.00E−05	L−lactate dehydrogenase | lldD
VC1332		2.34818	<5.00E−05	Tripartite tricarboxylate transporter TctA family
VCA0983		2.43475	<5.00E−05	L−lactate permease
VCA0227	*vctP*	2.47329	<5.00E−05	Ferric vibriobactin, enterobactin transport system, substrate−binding protein VctP
VC0091		2.48987	<5.00E−05	Uncharacterized SAM−dependent O−methyltransferase
VC1547−8	*exbB2* VC1548	2.54473	<5.00E−05	MotA/TolQ/ExbB proton channel family protein, TonB system biopolymer transport component; Chromosome segregation ATPase
VC0608	*fbpA*	2.57038	<5.00E−05	Ferric iron ABC transporter, iron−binding protein
VC0199		2.6252	<5.00E−05	Efflux ABC transporter, permease/ATP−binding protein Atu2242
VC1333		2.66616	<5.00E−05	Tripartite tricarboxylate transporter TctB family
VC0201−2−3	*fhuAC* VC0203	2.67288	<5.00E−05	Ferrichrome transport ATP−binding protein FhuC, Ferrichrome−binding periplasmic protein precursor, Ferrichrome transport system permease protein FhuB
VC1264	*irpA*	2.6755	<5.00E−05	Iron−regulated protein A precursor
VC1573	*fumC*	2.68696	<5.00E−05	Fumarate hydratase class II (EC 4.2.1.2) | fumC
VC2209	*vibF*	2.71161	<5.00E−05	Non−ribosomal peptide synthetase modules, siderophore biosynthesis | vibF
VCA0230	*vctC*	2.71848	<5.00E−05	Ferric vibriobactin, enterobactin transport system, ATP−binding protein
VC0474	*irgB*	2.842	<5.00E−05	Iron−regulated virulence regulatory protein irgB
VC2210	*viuB*	3.05349	<5.00E−05	Vibriobactin utilization protein ViuB
VCA0914	*hutB*	3.1391	<5.00E−05	Hemin ABC transporter, permease protein
VC2211	*viuA*	3.17699	<5.00E−05	Ferric vibriobactin receptor ViuA
VC0776−7−8	*viuPDG*	3.36327	<5.00E−05	Ferric vibriobactin, enterobactin transport system, substrate−binding protein ViuP, permease protein ViuD, permease protein ViuG
VCA0977		3.59744	<5.00E−05	ABC transporter, ATP−binding protein (cluster 5, nickel/peptides/opines)/ABC transporter, ATP−binding protein (cluster 5, nickel/peptides/opines)
VC0200	*fhuA*	3.62978	<5.00E−05	Ferrichrome−iron receptor
VCA0540	*focA*	3.73459	<5.00E−05	Formate efflux transporter FocA
VC0772	*vibE*	3.80191	<5.00E−05	2,3−dihydroxybenzoate−AMP ligase of siderophore biosynthesis @ 2,3−dihydroxybenzoate−AMP ligase
VC0771	*vibB*	4.12473	<5.00E−05	Isochorismatase of siderophore biosynthesis
VCA0907	*hutZ*	4.18631	<5.00E−05	Pyridoxamine 5’−phosphate oxidase−related putative heme iron utilization protein
VCA0908	*hutX*	4.24657	<5.00E−05	Putative heme iron utilization protein
VCA0909	*hutW*	4.29654	<5.00E−05	Radical SAM family protein HutW, similar to coproporphyrinogen III oxidase, oxygen−independent, associated with heme uptake
VC0775		4.40511	<5.00E−05	Amide synthase component of siderophore synthetase
VC1688	*mntX*	4.6546	<5.00E−05	Manganese transport protein MntX
VCA0576	*hutA*	4.70136	<5.00E−05	TonB−dependent heme and hemoglobin receptor HutA; TonB−dependent hemin, ferrichrome receptor
VCA0910−1−2	*tonB1* *exbB1* *exbD1*	4.74152	<5.00E−05	Putative TonB−dependent receptor, Ferric siderophore transport system, biopolymer transport protein ExbB, Biopolymer transport protein ExbD1
VCA0913		4.82235	<5.00E−05	Periplasmic hemin−binding protein
VC0774	*vibA*	4.90504	<5.00E−05	2,3−dihydro−2,3−dihydroxybenzoate dehydrogenase of siderophore biosynthesis @ 2,3−dihydro−2,3−dihydroxybenzoate dehydrogenase
VC0773	*vibC*	5.3735	<5.00E−05	Isochorismate synthase @ Isochorismate synthase of siderophore biosynthesis
VCA0233		5.66124	<5.00E−05	Hypothetical protein colocalized with Enterobactin receptor VctA
VBIVibCho83274_2864	*vctA*	8.67608	<5.00E−05	Enterobactin receptor VctA

ORF: open reading frame.

## Supplementary Materials

The following are available online at https://www.mdpi.com/2073-4425/11/10/1184/s1, Figure S1: Predicted structure of the product of VCA0231. (a) Predicted sequence and secondary structure of the protein encoded by VCA0231. The secondary structure was inferred by JPred available from Jalview. The results shown are the prediction using JNETPSSM and the jnetpred consensus using JNETPSSM, JNetALIGN and JNetHMM. Helices are indicated by red tubes and sheets by light green arrows. An AraC/XylS-type helix-turn-helix (HTH) domain with two HTH motifs detected using ScanProsite from ExPAsy (https://prosite.expasy.org/scanprosite/) is indicated. (b) Model of the tertiary structure. The predicted primary sequence of this protein was submitted to the web server of trRosetta (https://yanglab.nankai.edu.cn/trRosetta/) and a tertiary structure model was obtained and visualized using iCn3D from the NCBI (https://www.ncbi.nlm.nih.gov/Structure/icn3d/full.html). The helices are colored in red and sheets in green. The two HTH motifs are colored in blue. Figure S2: Functional association networks of *V. cholerae* VCA0231. Interaction network retrieved from STRING database for VCA0231. Nodes represent proteins and edges represent protein-protein interactions according to the types indicated in the figure. Table S1: Primers designed in this study, Table S2: Functional interactors predicted for VCA0231 from the STRING database.
