# Supplementary material for: IurV, Encoded by ORF VCA0231, Is Involved in the Regulation of Iron Uptake Genes in Vibrio cholerae
Source: Genes (Basel). 2020 Oct 12;11(10):1184. doi: 10.3390/genes11101184 (PMC7600106; doi:10.3390/genes11101184)
Supplement: Supplementary file 1 [file genes-11-01184-s001.pdf]

Figures and legends

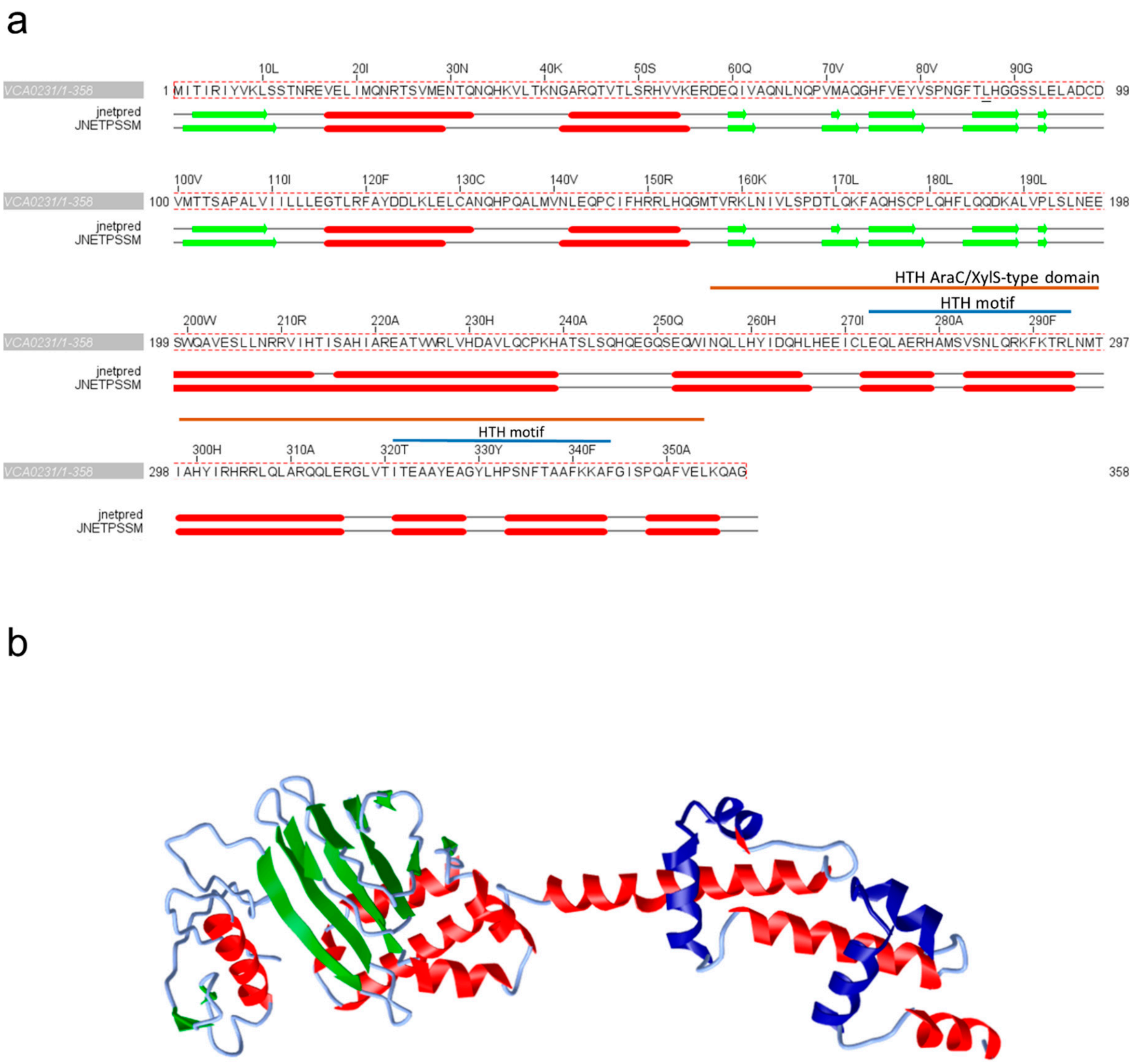

Supplemental Figure 1. Predicted structure of the product of VCA0231. (**a**) Predicted sequence and secondary structure of the protein encoded by VCA0231. The secondary structure was inferred by JPred available from Jalview. The results shown are the prediction using JNETPSSM and the jnetpred consensus using JNETPSSM, JNetALIGN and JNetHMM. Helices are indicated by red tubes and sheets by light green arrows. An AraC/XylS-type helix-turn-helix (HTH) domain with two HTH motifs detected using ScanProsite from ExPAsy (https://prosite.expasy.org/scanprosite/) is indicated. (**b**) Model of the tertiary structure. The predicted primary sequence of this protein was submitted to the web server of trRosetta (https://yanglab.nankai.edu.cn/trRosetta/) and a tertiary structure model was obtained and visualized using iCn3D from the NCBI (https://www.ncbi.nlm.nih.gov/Structure/icn3d/full.html). The helices are colored in red and sheets in green. The two HTH motifs are colored in blue.

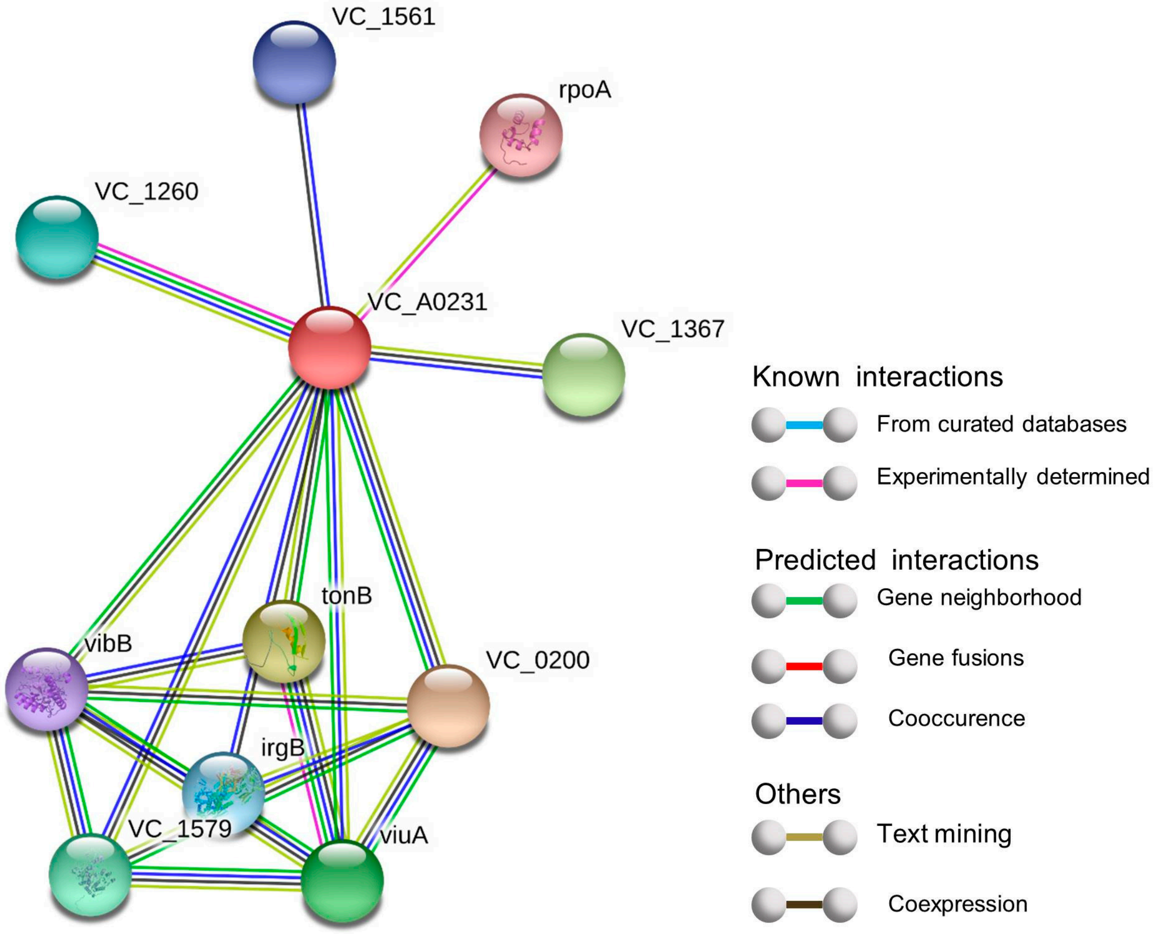

Supplemental Figure 2. Functional association networks of *V. cholerae* VCA0231. Interaction network retrieved from STRING database for VCA0231. Nodes represent proteins and edges represent protein–protein interactions according to the types indicated in the figure.

Supplemental Table 1. Primers designed in this study.

Primer	Sequence (5'-3')
araC H1P1	CCATACTGACTCAATTCCTCAAAAATACAAATGATAACGATTGGTGTAGGCTGG AGCTGCTTC
araC H2P2	AGGGGATAAGCTTGGCCAGAATCATGGCAATTAACCCGCCTGCATATGAATAT CCTCCTTAG
araC Fw	GCCATAACGCAGACTTCTTGC
araC Rv	GCTTATGTCTCTATACTCTTCC
fur Fw	GGTCTTAAAGTTACCCTCCCACG
fur Rv	GCCGCCTTCAAAATGGTGACG
SODFe Fw	GCCAAGCGATATTCATCCAAGG
SODFe Rv	GCTCAGTGGCCTATCTTCATGC
vctA Fw	CAGCACGCCAATATGTTCCA
vctA Rv	AGGCATAACCGCCGAAGATA
vctC Fw	GCCCATGAGCTGAATAAGGC
vctC Rv	GGCGCTCAACACTTCAGATT
**Primer**	**Sequence (5'-3')**

**araC H1P1**	CCATACTGACTCAATTCCTCAAAAATACAAATGATAACGATTGGTGTAGGCTGGAGCTGCTTC
**araC H2P2**	AGGGGATAAGCTTGGCCAGAATCATGGCAATTAACCCGCCTGCATATGAATATCCTCCTTAG
**araC Fw**	GCCATAACGCAGACTTCTTGC
**araC Rv**	GCTTATGTCTCTATACTCTTCC
**fur Fw**	GGTCTTAAAGTTACCCTCCCACG
**fur Rv**	GCCGCCTTCAAAATGGTGACG
**SODFe Fw**	GCCAAGCGATATTCATCCAAGG
**SODFe Rv**	GCTCAGTGGCCTATCTTCATGC
**vctA Fw**	CAGCACGCCAATATGTTCCA
**vctA Rv**	AGGCATAACCGCCGAAGATA
**vctC Fw**	GCCCATGAGCTGAATAAGGC
**vctC Rv**	GGCGCTCAACACTTCAGATT

Supplemental Table 2. Functional interactors predicted for VCA0231 from the STRING database.

Predicted functional partner		Neighborhood	Gene Fusion	Cooccurence	Coexpression	Experiments	Database	Textmining	Score
VC_0200	Iron(III) compound receptor	*		*	*			*	0.587
tonB	Protein TonB	*			*			*	0.503
VC_1367	GGDEF family protein			*	*			*	0.503
viuA	Vibriobactin receptor; Receptor for ferric vibriobactin	*		*				*	0.500
VC_1579	Enterobactin synthetase component F-related protein			*	*			*	0.498
VC_1260	Transcriptional regulator, AraC/XylS family	*		*		*		*	0.472
irgB	Iron-regulated virulence regulatory protein IrgB; Transcription activation of the irgA gene			*	*				0.465
VC_1561	Transcriptional regulator, LysR family			*	*				0.462
vibB	Vibriobactin-specific isochorismatase; Involved in the biosynthesis of the catechol siderophore vibriobactin	*			*			*	0.457
rpoA	DNA-directed RNA polymerase subunit alpha				*			*	0.454
